# Commentary on “The correlation between TRPV1 and pain during urethrocystoscopy: a prospective observational study”

**DOI:** 10.1097/JS9.0000000000003580

**Published:** 2025-09-30

**Authors:** Pengyu Hui, Xinyu Yao, Yonghui Du

**Affiliations:** Department of Urology, The Second Affiliated Hospital of Xi’an Medical University, Xi’an, Shaanxi, China

*Dear Editor,*

We read with keen interest the recently published article investigating TRPV1 expression in urine exfoliated cells and its correlation with pain and anxiety in patients undergoing urethrocystoscopy^[1]^. The authors’ initiative to explore the sensory molecule TRPV1 in the context of urology is innovative and potentially valuable. However, after a careful review of the study, we would like to raise several methodological and interpretative considerations that may guide future improvements.

First, although the identification of TRPV1 in exfoliated urinary cells is indeed novel, the biological rationale linking TRPV1 expression in urine to cystoscopy-induced pain requires further support. Previous studies have demonstrated the role of TRPV1 in bladder sensory function, but the choice to assess TRPV1 mRNA in exfoliated cells rather than mucosal biopsies or innervated tissues is only briefly addressed. The extent to which urinary TRPV1 mRNA reflects nociceptive signaling remains uncertain.

Second, regarding the detection of TRPV1 via RT-qPCR, the reported ΔCq difference between groups (~0.45; 9.48 ± 0.61 vs. 9.03 ± 0.72) reached statistical significance (*P* < 0.001), yet the biological and clinical relevance of such a small difference remains unclear. Moreover, no normalization was reported for epithelial cell content, RNA integrity, or urine concentration – factors that may introduce technical variability and confound interpretation.

Third, the authors utilized Least Absolute Shrinkage and Selection Operator (LASSO) regression for feature selection and subsequently constructed a logistic regression-based predictive model. However, critical metrics of model performance, such as area under the curve, calibration curves, or the Hosmer–Lemeshow goodness-of-fit test, were not provided. This omission limits the reader’s ability to assess the model’s discriminative power and clinical applicability. Additionally, the dichotomization of patients into “high” and “low” TRPV1 expression groups based on an unspecified cutoff lacks transparency and may increase the risk of type I error or outcome misclassification. Notably, while the difference in NRS scores between groups was statistically significant (*P* = 0.001), no significant difference was observed in Hamilton Anxiety (HAMA) scores (*P* = 0.77), yet the conclusion still implies a link with anxiety. This inconsistency weakens the rationale for suggesting TRPV1 antagonists as a potential anxiolytic intervention.

Fourth, the interpretation of causality warrants caution. Although the authors suggest a relationship between elevated TRPV1 expression and increased pain, the cross-sectional design does not support causal inference. Furthermore, factors such as benign prostatic hyperplasia and older age, both associated with TRPV1 expression and pain, may have introduced indication bias. A multivariable regression or mediation analysis could help elucidate potential confounding effects.

Fifth, the total sample size of 203 patients (with only 44 in the high-expression group) may have limited statistical power to detect differences in anxiety levels or explore subgroup interactions. This limitation may explain the non-significant results for HAMA scores.

In conclusion, the study proposes an intriguing hypothesis regarding the association between TRPV1 expression and procedural pain in urology. However, before TRPV1 can be considered a reliable biomarker or therapeutic target, further methodological refinement, external validation, and mechanistic investigation are necessary. We commend the authors for initiating this promising line of research and encourage future studies with more robust design and stronger clinical correlation. All of this content complies with the TITAN guidelines report^[2]^.

## Data Availability

Not applicable.
